# The effect of hamstring donor-site block for functional outcomes and rehabilitation after anterior cruciate ligament reconstruction

**DOI:** 10.3389/fsurg.2023.1003022

**Published:** 2023-01-25

**Authors:** Zijun Cai, Yuntao Yang, Di Liu, Wenhao Lu, Linyuan Pan, Miao He, Wenqing Xie, Dengjie Yu, Hengzhen Li, Hongfu Jin, Yusheng Li, Wenfeng Xiao

**Affiliations:** ^1^ Department of Orthopedics, Xiangya Hospital, Central South University, Changsha, China; ^2^ National Clinical Research Center for Geriatric Disorders, Xiangya Hospital, Central South University, Changsha, China

**Keywords:** anterior cruciate ligament, pain, post-operative rehabilitation, arthroscopy, femoral nerve block, local infiltration analgesia

## Abstract

**Purpose:**

To determine the effect of local infiltration anesthesia (LIA) at the donor site combined with a femoral nerve block (FNB) on short-term postoperative pain, functional outcomes, and rehabilitation after arthroscopic hamstring tendon autograft anterior cruciate ligament reconstruction (ACLR).

**Methods:**

This study was a single center, randomized controlled trial. Seventy-three subjects with ACL rupture were enrolled. Participants were randomly allocated to two groups, 47 in the experimental group (Group A) and 26 in the control group (Group B). All operations were performed under FNB. In Group A, 10 ml of 1% ropivacaine was injected precisely at the hamstring donor site. Patients in Group B were treated with the same amount of saline. Preoperatively and postoperatively, pain scores based on the numerical rating scale (NRS) and consumption of opioids were recorded. In addition, knee functions were assessed by the International Knee Documentation Committee Subjective Knee Form (IKDC), the Lysholm score, and the Knee injury and Osteoarthritis Outcome Score (KOOS) preoperatively and postoperatively at 1 and 3 months. In addition, we applied the KNEELAX3 arthrometer to evaluate the stability of the knee preoperatively and postoperatively so that subjective and objective knee conditions were obtained to help us assess knee recovery in a comprehensive manner.

**Results:**

The hamstring donor-site block reduced pain within the first 12 postoperative hours. There were no significant differences between two groups in pain intensity preoperatively and equal to or greater than 24 hours postoperatively. Furthermore, there were no differences between the groups concerning knee functions preoperatively or in the short-term follow-up at 1 and 3 months.

**Conclusion:**

LIA at the donor site can effectively improve the early postoperative pain of patients after ACLR and reduce the use of opioids without affecting the functional outcomes of the surgery.

## Introduction

ACL injuries may account for 50% of sports-related injuries in high school athletes (1). Although there is not a single epidemiological survey that summarizes populations around the world, we can be certain, based on several local studies, that the incidence rate of ACL injuries is high worldwide. In the United States, it affects more than two hundred thousand people each year, with direct and indirect costs greater than $7 billion annually (1). In Sweden, the overall incidence of ACL injury was 78 per 100,000 (2). In Finland, the total ACL injury incidence was about 23.3 per 100,000 person-years (3). Currently, ACLR is standard practice for athletes that wish to return to high-level activities. Hamstring tendon graft ACLR is one of the most common and effective orthopedic procedures. The all-inside technique (AIT) for ACLR is becoming increasingly popular because of its anatomical adaptability, low invasiveness, and rapid rehabilitation (4).

With the progress of modern medicine, the patient's comfort during the treatment process has been continuously considered. The pain has been recognized the fifth vital sign and it has received increasing attention in clinical diagnosis and treatment practice (5). Pain is one of the prominent complaints in patients after ACLR. Compared with other types of orthopedic sports surgery, ACLR causes not only more significant but also longer-lasting pain to patients, often throughout the entire rehabilitation process (6). Reasonable pain management can improve outcomes and a higher pain is often associated with more difficulties during rehabilitation, even in daily activities (7). Pain, together with other factors, are related to knee function.

A reasonable perioperative analgesia approach is necessary for faster rehabilitation and satisfaction. Currently, intravenous or oral opioids are frequently prescribed for postoperative pain control following ACLR (8). However, if the use of these drugs is not strictly controlled, it may not only increase the risk of various complications but also increase the possibility of drug dependence, abuse, and overdose (8). In the USA, it is estimated that opioid-related complications cost $78.5 billion every year (8, 9). There were approximately 49,680 deaths caused by opioid overdose in 2019, accounting for 70.6% of all drug overdose deaths (8, 10). There has been an increasing call to strengthen the management of opioid use and to take into account their possible harm to patients (8, 10). It is urgent to develop a novel and suitable approach to reduce postoperative pain in patients after ACLR that does not rely on opioids.

According to our clinical experience, the main sources of pain after ACLR include the following three points: the skin incision, the graft fixation positions, and the graft donor site. The former two cannot be avoided, so we focused on relieving the pain at the donor site. FNB has been shown to be an effective method for relieving postoperative pain (11). We believe that combining analgesia at the donor site with FNB may result in better outcomes. To date, there have been several relevant studies in favor of our perspective (12–15). However, few studies have compared the effect of injecting or not injecting local anesthesia to perform donor-site block on the postoperative condition of patients. With this study, we hope to fill the gap in knowledge in this area.

Pain assessment is usually derived from subjective statements rather than objective measurements (16). Thus, it is conceivable that the same level of pain may be described as different subjective feelings by different individuals. This problem does exist, but few scholars have suggested that it needs to be addressed in regard to pain-related research. To obtain a more objective pain assessment, a new preoperative evaluation program was introduced. The cold pressor test (CPT) is a research tool that can induce pain perception in humans (17). In this way, we were able to obtain the pain threshold and pain tolerance of each patient before the operation, which helped eliminate the influence of individual differences in pain perception on the study findings (17).

We aimed to determine the effect of LIA at the donor site combined with a FNB on short-term postoperative pain and the functional outcomes and rehabilitation after arthroscopic hamstring tendon autograft ACLR. We hypothesized that the combination of FNB and LIA can achieve better analgesic effect, but it is not enough to directly impact the postoperative rehabilitation speed.

## Materials and methods

This research was designed as a randomized controlled trial. Patients were designed to be randomized to undergo either a hamstring donor-site block or isotonic sterile water of equal volume, in an around 2:1 ratio. A permuted block randomization scheme was used with block sizes of 3. All operations were performed by the same experienced orthopedic surgeon in our hospital. The study protocol was approved by the institutional ethics committee. Informed consent for participation in the study was obtained from all patients before randomization.

### Patients

**Figure 1:**
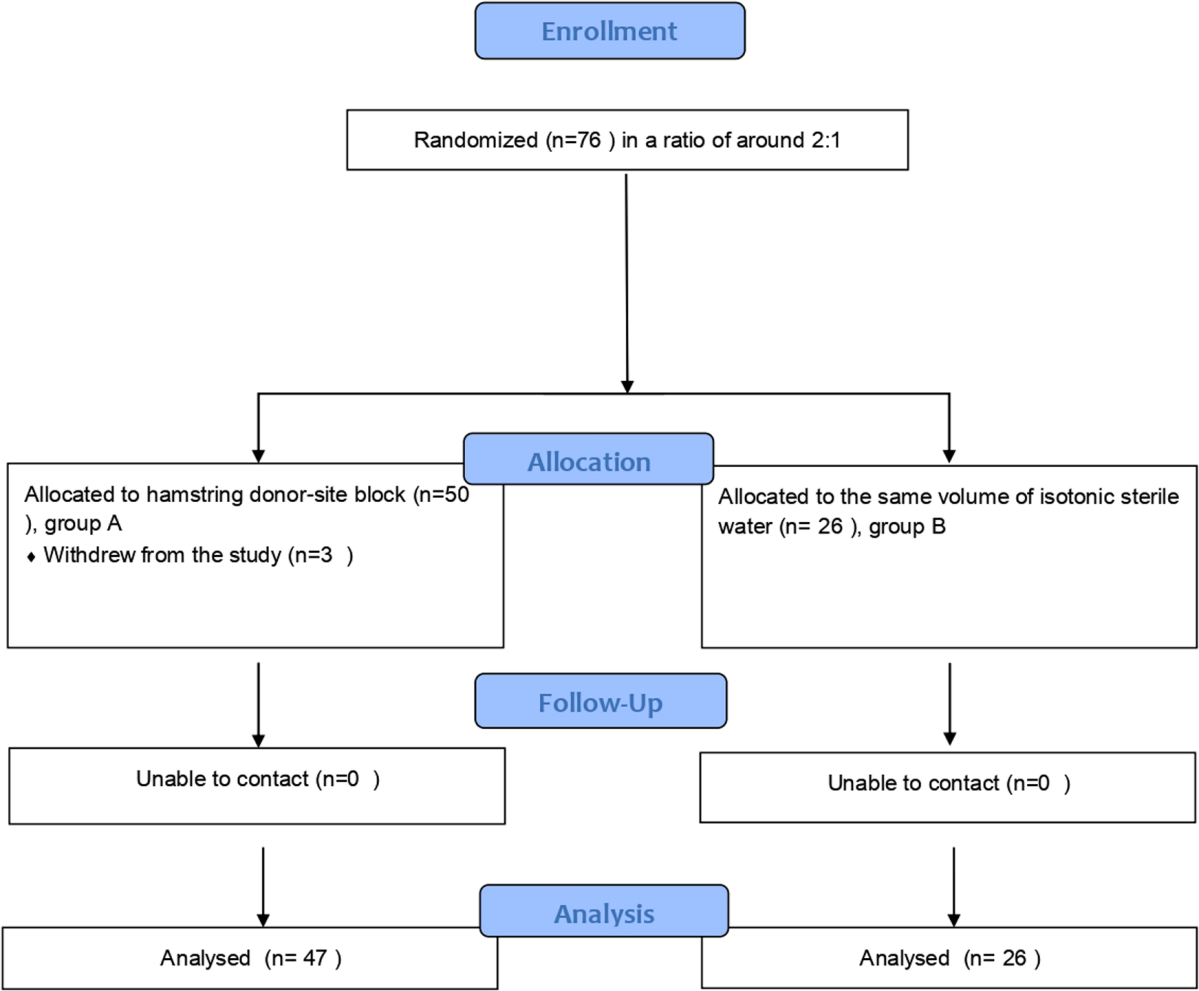
Flowchart of study participants.

Patients 15–50 years of age who were diagnosed with ACL rupture, usually confirmed by means of history, physical examination, and magnetic resonance imaging, were screened for eligibility. Participants were randomly allocated to two groups (Figure 1). Baseline data were collected. A total of 73 (originally 76) patients with an American Society of Anesthesiologists (ASA) physical status classification of I–III who satisfied the inclusion criteria of our study underwent arthroscopic ACLR using hamstring tendon autografting for ACL tears. Among them, 47 (excluding 3 withdrawing from the study) patients in Group A were given a 1% 10 ml ropivacaine injection right at the donor site, while the other 26 patients in Group B were given the same volume of saline.

The inclusion criteria were primary ACLR and a healthy contralateral knee. Exclusion criteria included refusal of surgical treatment; severe concomitant injuries; concurrently performed other different surgical procedures; weight less than 35 kg; body mass index (BMI) greater than 45 kg/m^2^; ASA physical class greater than 4; allergy to local anesthetics; preexisting neurologic deficit; undergoing revision surgery; any opioid use within the previous 3 months; inability to understand the NRS; or suffering from serious or chronic conditions that may have an impact on surgical treatment, postoperative recovery or regular follow-up. After returning to the ward after surgery, tramadol was orally administered if the pain at rest exceeded 3 on NRS. A unified analgesic scheme and postoperative rehabilitation plan were adopted during the perioperative period.

### Evaluations

The IKDC, the Lysholm score, and the KOOS were used to examine the functional outcomes preoperatively, at the 1-month follow-up, and at the 3-month follow-up. In addition, the degree of anterior tibial translocation was measured with a KNEELAX3 arthrometer (Monitored Rehab Systems, Haarlem, The Netherlands) (18) preoperatively, at the 1-month follow-up, and at the 3-month follow-up. As KT-1000/2000, it was also considered valid and reliable, with very similar operating process (19, 20). Both knees were measured with different anterior force with the maximum of 132 N applied to the proximal tibia at 20° of knee flexion in a supine position. Actually, this helped quantify the Lachman test as a result. The difference in the degree of anterior translocation between the nonaffected side and affected side was expressed as in millimeters (18). To minimize any susceptibility bias, all evaluations were performed by the same person. According to the KNEELAX3 instructions, it is recommended that patients be measured within grade 3 within 6 months after surgery.

Pain management success was assessed by the consumption of opioids (tramadol) during the patient's hospitalization and pain scores on an NRS, an 11-point scale where 0 indicates no pain and 10 indicates the worst imaginable pain (21), which is considered comparable to the visual analog scale (VAS) (22). Opioids were provided only if the patient made a strong request and the NRS score was higher than 3. Patients were asked to give a score based on NRS preoperatively and at 2, 4, 6, 12, 24, 48 h, 1 month, and 3 months postoperatively. CPT was performed before surgery.

### Surgical technique

AIT for ACLR is becoming increasingly popular and it has been described extensively in previous papers (23, 24). Here, only those parts relevant to the research topic are retained.

**Figure 2:**
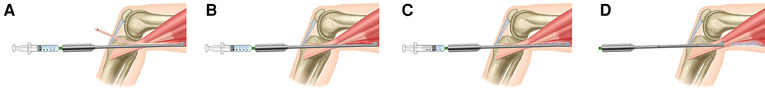
Precise donor-site block steps; (**A**) ready to retrieve tendon, (**B**) tendon removed, (**C**) ropivacaine injected right at the deepest point of tendon removal site, (**D**) the whole tendon removal site full of ropivacaine.

All patients received a preoperative FNB using an ultrasound-guided technique. One of the highlights of this study is the process of harvesting the hamstring tendon and subsequent local anesthesia. The medial tibial tuberosity incision was made where the hamstring tendon is removed with our self-developed tendon retriever. The surgeon initially obtained the tendon, while not moving the retriever, and slowly inject the 10 ml of ropivacaine. Then, the analgesic continued to be injected while withdrawing the retriever, and finally, the ropivacaine would fill the entire donor site (Figure 2).

### A brief introduction to our novel tendon retriever

**Figure 3:**
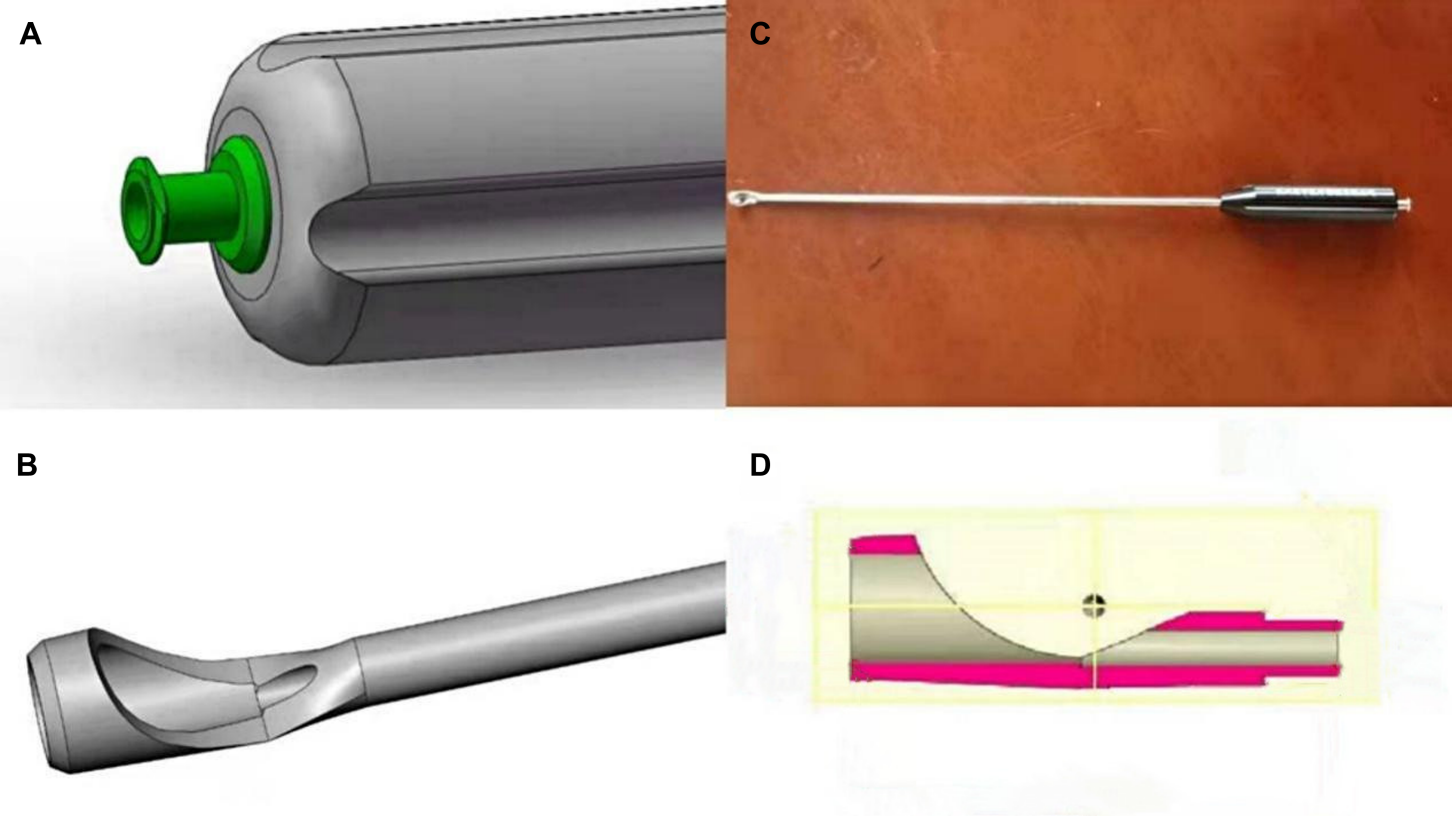
Our novel tendon retriever; (**A**) end of tendon retriever, (**B**) top of tendon retriever, (**C**) overall view, (**D**) internal structure..

We designed a novel tendon retriever capable of precisely injecting local anesthetics, as shown in Figure 3. The outer tube body has a hollow channel and a positioning wire perforation along the axis and a plurality of anesthesia channels extending along the axis and with outlets located at different heights of the outer tube body. The inner end of the tendon collection rod body has an opening ring that can form a closed ring with the outer tube body, and the tendon collection rod body has a penetration hole extending along the axis. The inner end of the positioning member has a C-shaped positioning ring, a flexible cross-file is provided inside the C-shaped positioning ring. One set of rings is fixed to the C-shaped positioning ring, and the other set of rings is set on the C-shaped positioning ring. The restriction structure set in the perforation of the positioning wire is used to clamp or release the positioning member, and the C-shaped positioning ring is fixed to the end of the positioning rod.

### Cold pressor test

The CPT was first created and utilized as a tool by Edgar A. Hines, Jr. to study blood pressure variability (17). During the test, the patient needs to put the whole palm of their hand into cold water that is constantly circulating through ice. Since this test is based on hemodynamic response to peripheral cold stimulation, it doesn't matter which hand people would use. The experience is made more painful by the presence of the circulating pump that prevents the buildup of a warmer layer of water around the forearm. With the extension of time, the pain in the palm of the hand will become more intense until the patient cannot tolerate it and pulls their hand out of the water. Previous studies have shown that the CPT can be used to help surgeons better cope with pain problems in patients (25). CPT can be used not only to predict the degree of postoperative pain, but also to help standardize the obtained pain data (25).

The general flow of this test is as follows. A bucket full of cold water with ice should be prepared to keep the temperature constant. The water temperature was monitored (maintained at 4°C), as a 2°C difference in temperature can affect tolerance time and pain intensity (25). In addition, the bucket needs to be equipped with a device that allows the water to circulate continuously to avoid the water temperature in the area near the palm of the hand being slightly higher than the preset water temperature. In a word, as the ice melted, the surface temperature of the water was maintained, and in addition the circulating pump could make the temperature anywhere in the water the same. The participant would be briefed by the observer on how to take the test in advance. The patient will be asked to extend their hand into the ice water to ensure that the water floods the straight wrist transverse striae while the observer starts timing. The participant informs the observer immediately when their hand sensation changes from cold to pain, and the time difference between the beginning of the test and the first report of pain is recorded as that participant's “pain threshold” (25, 26). They are instructed to voluntarily withdraw their hand at the point at which the pain becomes “unbearable”—the time between the beginning of the test and this voluntary withdrawal is recorded as that participant's “pain tolerance” (25, 26). To ensure safety, the test is forcibly terminated if the participant does not remove their hand within 3 min.

### Postoperative rehabilitation

Within 1 week after surgery, complete knee extension was required during bed rest, quadriceps contraction and straight leg raising training were performed, ankle flexion and extension activities were performed, and passive knee flexion was performed. Partial weight-bearing with crutches when walking on the ground; passive flexion of the knee joint to 90 degrees was started on the 8th day, and full weight-bearing standing and walking with detached crutches were trained; at the end of the 2nd week, passive flexion of the knee joint to 120 degrees and full weight-bearing walking were required; fixed bicycle exercises, terminal extension and balance exercises were performed at 3–6 weeks; at 7–12 weeks, active and passive knee flexion, range of motion consistent with the unaffected side, knee extension and resistance knee extension, and full squatting were performed. Full return to full activities of daily living started 3 months and gradual return to sports started 9 months.

### Statistical analysis

Calculation of the sample size was based on an expected difference of two points on the NRS score between the groups. With a power of 0.80 and *α* = 0.05, a sample size of at least 25 patients per group was obtained. SPSS 26.0 software was used for statistical analysis of the data. Measures were tested for normality using the D'Agostino test, and normally distributed indicators are expressed as the mean ± standard deviation (SD) and compared between groups using the independent samples *t*-test; nonnormally distributed indicators are expressed as the median and interquartile range (IQR) and compared between groups using the Mann–Whitney *U* test. Multifactorial logistic regression analysis was performed to assess the correlation between NRS scores and local anesthesia or not within 48 h preoperatively and postoperatively after adjusting for confounders. Statistical data are expressed as percentages (%), and comparisons between groups were made by the chi-square test or Fisher's exact probability method. *P* < 0.05 was considered a statistically significant difference.

## Results

From 2020 to 2022, 73 patients were included in this study. The baseline characteristics were similar between the two groups concerning gender, age, BMI, time from injury to hospital admission, pain tolerance, and intraoperative tourniquet application time (Table 1). However, the pain threshold was higher in the LIA group (55.87 ± 6.18 vs. 50.85 ± 4.42, *P* < 0.001). Within the first 12 h after surgery, pain level was significantly lower in Group A than in Group B (*P* < 0.05). There was no difference in pain scores preoperatively and at the time point greater than or equal to 24 h postoperatively (Table 2), but the consumption of opioids during the patient's hospitalization showed a significant difference between groups (*P* < 0.05) (Table 3). Since we collected objective data on pain perception (CPT), so standardizing the scores of the NRS based on pain perception could be considered. After regression analysis, there was no change in the conclusion in terms of pain (Table 4). This study showed no differences in functional outcomes between groups at all follow-up time points (Tables 5–7). No complications related to the anesthesia or nerve block were observed.

**Table 1:** Patient characteristics (*n* = 73).

	Group A (*n* = 47)	Group B (*n* = 26)	*T*/*U*/*c*^2^	*P* value
Gender			1.780	0.182
Male	31 (66.0%)	13 (50.0%)		
Female	16 (34.0%)	13 (50.0%)		
Age (years)	23.00 (20.00–25.00)	24.50 (20.75–26.25)	737.000	0.145
Time from injury to hospital admission (days)	30.00 (7.00–150.00)	30.99 (11.75–157.50)	670.500	0.492
Pain threshold (seconds)	55.87 ± 6.18	50.85 ± 4.42	3.656	<0.001
Pain tolerance (seconds)	107.72 ± 6.53	109.19 ± 7.40	−0.877	0.383
BMI (kg/m^2^)	22.40 (21.30–23.90)	23.50 (21.27–25.02)	733.500	0.158
Tourniquet time (minutes)	82.00 (79.00–86.00)	83.00 (79.00–87.00)	655.000	0.610

**Table 2:** Patient-reported NRS.

	Group A	Group B	*T* value	*P* value
NRS (preoperatively)	3.06 ± 1.90	3.15 ± 1.22	−0.245	0.807
NRS (2 h postoperatively)	2.62 ± 1.69	3.77 ± 1.86	−2.692	0.009
NRS (4 h postoperatively)	2.98 ± 1.46	4.23 ± 1.82	−3.203	0.002
NRS (6 h postoperatively)	3.87 ± 1.20	4.96 ± 1.54	−3.343	0.001
NRS (12 h postoperatively)	5.55 ± 1.21	6.54 ± 1.27	−3.222	0.002
NRS (24 h postoperatively)	3.89 ± 1.31	4.12 ± 1.18	−0.719	0.475
NRS (48 h postoperatively)	3.04 ± 1.08	3.12 ± 1.07	−0.276	0.783

**Table 3:** Opioids consumption.

	Group A (*n* = 47)	Group B (*n* = 26)	*U*	*P* value
Opioids consumption (mg)	50.00 (40.00–60.00)	60.00 (47.50–72.50)	905.500	0.001

**Table 4:** Regression analysis of postoperative NRS.

NRS	*β*	SE	*t*	*P*	*β*	95% CI
2 h	−0.930	0.465	−2.000	0.049	−0.246	−1.858 to −0.003
4 h	−0.965	0.420	−2.296	0.025	−0.274	−1.804 to −0.127
6 h	−0.915	0.354	−2.585	0.012	−0.310	−1.621 to −0.209
12 h	−0.798	0.326	−2.447	0.017	−0.293	−1.449 to −0.148
24 h	−0.085	0.336	−0.253	0.801	−0.033	−0.755 to 0.586
48 h	0.055	0.287	0.193	0.848	0.025	−0.517 to 0.627

**Table 5:** NRS, IKDC, lysholm, KOOS, and KNEELAX evaluation preoperatively.

	Group A	Group B	*T*/*U*	*P* value
NRS	3.06 ± 1.90	3.15 ± 1.22	−0.245	0.807
IKDC	51.68 ± 12.65	50.77 ± 13.03	0.292	0.771
Lysholm	54.47 ± 21.31	50.27 ± 14.49	0.895	0.374
**KOOS**				
Symptoms	61.96 ± 18.64	63.96 ± 12.58	−0.489	0.626
Pain	69.91 ± 13.77	70.27 ± 10.09	−0.115	0.909
Function in daily living	79.81 ± 10.30	80.27 ± 7.61	−0.200	0.842
Sports/recreation	47.55 ± 25.72	48.46 ± 26.06	−0.144	0.886
Quality of life	31.79 ± 10.68	33.08 ± 12.55	−0.464	0.644
**KNEELAX (mm)**				
Affected side	6.69 ± 1.19	6.55 ± 1.41	0.432	0.667
Healthy side	1.80 ± 0.50	1.79 ± 0.51	0.113	0.910

**Table 6:** NRS, IKDC, lysholm, KOOS, and KNEELAX evaluation 1 month postoperatively.

	Group A	Group B	*T*/*U*	*P* value
NRS	2.17 ± 1.47	2.58 ± 1.77	−1.048	0.298
IKDC	81.02 ± 12.56	81.42 ± 14.70	−0.123	0.902
Lysholm	81.02 ± 5.82	81.88 ± 5.53	−0.626	0.534
**KOOS**				
Symptoms	81.70 ± 7.61	83.38 ± 5.86	−0.977	0.332
Pain	82.28 ± 6.56	83.50 ± 7.30	−0.733	0.466
Function in daily living	83.85 ± 9.16	85.69 ± 7.10	−0.886	0.378
Sports/recreation	81.91 ± 10.19	83.85 ± 6.67	−0.867	0.389
Quality of life	81.26 ± 8.67	81.81 ± 9.18	−0.255	0.799
Pain improvement (cm)	8.00 (7.50–8.40)	7.70 (7.50–8.20)	533.500	0.371
Quality of life improvement (cm)	7.70 (7.20–8.20)	7.50 (7.37–7.70)	496.500	0.186
**KNEELAX (mm)**				
Affected side	1.74 ± 0.49	1.84 ± 0.48	−0.845	0.401
Healthy side	1.81 ± 0.50	1.78 ± 0.50	0.247	0.806

**Table 7:** NRS, IKDC, lysholm, KOOS, and KNEELAX evaluation 3 months postoperatively.

	Group A	Group B	*T*/*U*	*P* value
NRS	1.68 ± 1.23	1.69 ± 1.56	−0.034	0.973
IKDC	83.89 ± 11.08	85.69 ± 13.30	−0.618	0.539
Lysholm	83.68 ± 5.17	85.81 ± 5.72	−1.619	0.110
**KOOS**				
Symptoms	84.89 ± 6.67	86.58 ± 5.42	−1.100	0.275
Pain	86.19 ± 7.45	87.42 ± 6.28	−0.713	0.478
Function in daily living	89.74 ± 5.59	90.38 ± 5.36	−0.475	0.636
Sports/recreation	87.77 ± 6.74	88.85 ± 4.75	−0.723	0.472
Quality of life	86.60 ± 7.51	87.92 ± 5.00	−0.806	0.423
Pain improvement (cm)	8.30 (8.00–8.60)	8.10 (7.90–8.50)	471.500	0.106
Quality of life improvement (cm)	8.00 (7.60–8.40)	8.05 (7.47–8.40)	550.000	0.481
**KNEELAX (mm)**				
Affected side	1.79 ± 0.49	1.63 ± 0.51	1.330	0.188
Healthy side	1.84 ± 0.48	1.75 ± 0.50	0.740	0.462

## Discussion

The most important finding of this study was a statistically significant reduction in postoperative pain scores with the additional use of donor-site block. The significant complications of ACLR consist of infection, hematoma or hemarthrosis, failure of the surgery, etc. (27). However, based on our experience in clinical practice, cases most commonly seen contain quadriceps weakness, restriction of knee flexion or extension, and donor-site or incision pain. Pain can often lead to the exacerbation of the first two issues. Not only is pain subjectively troubling for the patient, but it can also objectively hinder rehabilitation efforts, lead to a decreased range of motion, and have an overall negative effect on surgical outcomes (14).

To date, few studies have been conducted on the effect of LIA at the donor site combined with FNB on short-term postoperative pain and functional outcomes after arthroscopic hamstring tendon autograft ACLR. The reduction of pain level was obvious within 12 hours after ACLR but this effect was no longer working beyond the period. In addition, the additional donor site block after FNB did not affect any other subjective or objective outcomes. However, an assumption should not be made arbitrarily that donor-site LIA did not have any positive effects on functional recovery since the first assessment of function was at 1 month postoperatively. Less pain is often accompanied by a greater range of motion. Additional block could provide comfort to the affected region and a greater range of motion during the immediate postoperative course.

The study we conducted was similar to Bushnell et al. (14) in 2010, and they found a remarkable decrease in VAS score among patients treated with both LIA and FNB compared with FNB alone. Our study makes the same conclusions. However, their study may be associated with some bias because of the absence of opioid consumption records, an insufficient follow-up period, and a lack of baseline pain scores preoperatively. Therefore, we assessed preoperative pain scores as well as the pain threshold and pain tolerance, extended the follow-up time, recorded opioid consumption, and further took into consideration subjective and objective functional outcomes to improve the study design to eventually make a more comprehensive evaluation of the effect of a donor site block. Another highlight of our study is the utilization of the novel tendon retriever that we invented, which ensures precise anesthesia at the donor site. As the core variable in this study, it deserves to be made as accurate as possible. The tools and methods for implementing LIA have not been harmonized between different studies, which may have an impact on the correctness of the trial conclusions. Previous similar studies have always ignored this point.

Considering that gender is one of the confounders of pain (28), the baseline characteristics of the subjects were analyzed. The slight baseline difference between the two groups probably did not bias our results.

The best choice of anesthetic for local infiltration has not yet been determined, with bupivacaine and ropivacaine being commonly used. Bupivacaine has a half-life of 3.5 h, whereas ropivacaine is a long-acting local anesthetic with a similar structure to bupivacaine and a half-life of 4.2 h. Ropivacaine has the advantage of being less toxic, and its blockade of sensation is stronger than its block against movement (29), which is why we selected it.

It was expected that the range of motion would be highly associated with pain intensity. Based on our observations of postoperative patients, patients with less pain did have a greater knee range of motion and were more comfortable moving their knees. Therefore, range of motion was not specifically described in this study.

Less early postoperative pain could be observed without the use of a tourniquet during routine knee arthroscopy (30), suggesting the potential impact of the application of a tourniquet intraoperatively on postoperative rehabilitation, but some studies have noted that a tourniquet use of less than approximately 2 h had no effect on the strength of the lower extremity and pain scores (31, 32). There was no significant difference in the mean tourniquet times between the two groups in this study, so this potential interfering factor was eliminated. It is worth noting that Guler et al. (33) showed that a local anesthetic injection is more beneficial after tourniquet release. Thus, future studies may consider investigating the timing of the injection as a factor in pain control.

Several papers previously reported an abnormal sensation in the lower extremities after surgery. This complication often occurs because of injury to the lateral sural cutaneous or saphenous nerve due to the incision or intraoperative retraction (14). In our study, there were no neurological problems related to the use of the donor-site injection technique.

There are some limitations of this study. There was no intentional blinding of patients, doctors, or nurses in the postanesthesia care unit. Moreover, patient satisfaction during the whole course, speed of recovery, and the immediate postoperative condition of the body and knee could not be presented merely according to our study data.

## Conclusion

The simplicity of the donor-site block technique warrants its extensive application. It could effectively improve the early postoperative pain of patients after ACLR and reduce the use of opioids, without affecting follow-up functional outcomes. Future related studies with larger samples may help confirm its beneficial effect.

## Data Availability

The data supporting the findings of this study are available from the corresponding author, upon reasonable request.
